# Genome-Wide Identification of the *14-3-3* Gene Family and Its Involvement in Salt Stress Response through Interaction with NsVP1 in *Nitraria sibirica* Pall

**DOI:** 10.3390/ijms25063432

**Published:** 2024-03-19

**Authors:** Xihong Wan, Rongfeng Duan, Huaxin Zhang, Jianfeng Zhu, Haiwen Wu, Huilong Zhang, Xiuyan Yang

**Affiliations:** 1 Institute of Ecological Conservation and Restoration, Chinese Academy of Forestry, Beijing 100091, China; 13659034689@163.com (X.W.); drfeng928@163.com (R.D.); zhanghx1998@126.com (H.Z.); jfzhu@caf.ac.cn (J.Z.); auhheaven@163.com (H.W.); 2 Tianjin Institute of Forestry Science, Chinese Academy of Forestry, Tianjin 300457, China; 3 Comprehensive Experimental Center of Chinese Academy of Forestry in Yellow River Delta, Dongying 257500, China

**Keywords:** *14-3-3* gene family, *N. sibirica*, *NsVP1*, salt tolerance

## Abstract

14-3-3 proteins are widely distributed in eukaryotic cells and play an important role in plant growth, development, and stress tolerance. This study revealed nine *14-3-3* genes from the genome of *Nitraria sibirica* Pall., a halophyte with strong salt tolerance. The physicochemical properties, multiple sequence alignment, gene structure and motif analysis, and chromosomal distributions were analyzed, and phylogenetic analysis, cis-regulatory elements analysis, and gene transcription and expression analysis of *Ns14-3-3s* were conducted. The results revealed that the *Ns14-3-3* gene family consists of nine members, which are divided into two groups: ε (four members) and non-ε (five members). These members are acidic hydrophilic proteins. The genes are distributed randomly on chromosomes, and the number of introns varies widely among the two groups. However, all genes have similar conserved domains and three-dimensional protein structures. The main differences are found at the N-terminus and C-terminus. The promoter region of *Ns14-3-3s* contains multiple cis-acting elements related to light, plant hormones, and abiotic stress responses. Transcriptional profiling and gene expression pattern analysis revealed that *Ns14-3-3s* were expressed in all tissues, although with varying patterns. Under salt stress conditions, *Ns14-3-3 1a*, *Ns14-3-3 1b*, *Ns14-3-3 5a*, and *Ns14-3-3 7a* showed significant changes in gene expression. *Ns14-3-3 1a* expression decreased in all tissues, *Ns14-3-3 7a* expression decreased by 60% to 71% in roots, and *Ns14-3-3 1b* expression increased by 209% to 251% in stems. The most significant change was observed in *Ns14-3-3 5a*, with its expression in stems increasing by 213% to 681%. The yeast two-hybrid experiments demonstrated that Ns14-3-3 5a interacts with NsVP1 (vacuolar H^+^-pyrophosphatase). This result indicates that Ns14-3-3 5a may respond to salt stress by promoting ionic vacuole compartmentalization in stems and leaves through interactions with NsVP1. In addition, *N. sibirica* has a high number of stems, allowing it to compartmentalize more ions through its stem and leaf. This may be a contributing factor to its superior salt tolerance compared to other plants.

## 1. Introduction

14-3-3 proteins are expressed in a wide range of organisms and tissues, indicating their significance and multifunctionality [1]. In plant cells, 14-3-3 proteins are considered to be nodes in signaling networks and the core of interactions between proteins [1,2]. More than 300 target proteins have been reported to interact with the 14-3-3 proteins [3]. The 14-3-3 proteins are composed of two monomers, each of which contain antiparallel α-helices, and the monomers bind to each other through their N-termini to form homo- or heterodimers [4,5]. This formation provides internal channels that can bind to phosphorylated target proteins. The formation of various dimers by a large number of isomers forms the basis for 14-3-3 proteins to perform complex functions. Each monomer of the dimer can bind to a target protein, indicating that the 14-3-3 protein can interact with two target proteins simultaneously [4,6]. 14-3-3 proteins specifically recognize phosphorylated target proteins and regulate protein–protein interactions in a phosphorylation-dependent manner [5,7]. They thereby exert signal transduction functions and participate in the regulation of many biological processes.

14-3-3 proteins in plants are involved in various processes such as cell elongation, division, seed germination, vegetative and reproductive growth, seed dormancy, protein kinase regulation, splicing events, light and hormone signals, metabolism, material transport, and cell cycle regulation [2,8]. In addition, 14-3-3 proteins play an important role in plant resistance to abiotic stresses such as salt stress [2,8]. The expression of the *14-3-3* genes was observed to increase following exposure to salt in many species, e.g., *OsGF14b-OsGF14g* in *Oryza Sativa* [9] and *Gh14-3-3b* and *Gh14-3-3c* in *Gossypium hirsutum* roots [10]. It has also been shown that overexpression of the *14-3-3* genes can enhance salt tolerance. The salt tolerance of *Arabidopsis* plants can be enhanced by overexpressing the tomato *TFT7* gene [11]. Similarly, overexpressing the *Brachypodium distachyon BdGF14d* gene in tobacco led to improved salt tolerance [12]. The 14-3-3 protein may regulate salt stress by interacting with salt-responsive proteins. In *Phaseolus vulgaris*, PvGF14g and PvGF14a can interact with PvSOS2 and may be involved in the regulation of salt tolerance by interacting with PvSOS2 [13]. In *Arabidopsis*, 14-3-3λ and 14-3-3κ modulate plant salt tolerance by inhibiting the SOS pathway through inhibition of SOS2 activity [14]. The 14-3-3 protein GRF6 interacts with phosphorylated vacuolar two-pore K^+^ channel 1 (TPK1) to enhance TPK1 activity [15,16]. This promotes potassium efflux into the cytoplasm and enhances salt tolerance. Furthermore, 14-3-3ω interacts with H^+^-ATPase, thereby activating its activity to transport protons out of the cell and establish a transmembrane proton gradient for SOS1 [17,18]. All these results suggest that 14-3-3 proteins may be involved in the regulation of salt tolerance in plants by interacting with other proteins. Genome-wide characterization of 14-3-3 proteins in *Arabidopsis* [19], rice [20], tomato [21], and mango [22] has been carried out, and they can be organized into the ε and non-ε groups. However, the *14-3-3* gene family in *N. sibirica* has been not characterized as of yet.

*N. sibirica* is a dicotyledonous shrub that belongs to *Nitraria L*. which is widely used in landscape beautification and ecological restoration of saline-alkali land. It is a typical halophytic plant that can survive extreme drought and saline environments, with great salt tolerance and environmental adaptability making it a nice model for studying the role of 14-3-3 proteins in stress tolerance [23,24,25]. Accordingly, in this study, we identified nine members of the *Ns14-3-3* gene family from the genomic data of *N. sibirica*. The physicochemical properties, chromosomal localization, gene structure, evolutionary relationships, promoter cis-regulatory elements, and expression patterns in different tissues and in response to salt stress treatments were analyzed.

Vacuolar H^+^-pyrophosphatase (V-H^+^-PPase) is a crucial enzyme that exists on the tonoplast to maintain pH homeostasis across the vacuolar membrane. This enzyme generates a proton gradient between the cytosol and a vacuolar lumen by hydrolysis of a metabolic byproduct, pyrophosphate (PPi) [26]. A variety of ion transporters on the vacuolar membrane under salt stress can use the H^+^ gradient generated by V-H^+^-PPase to transport ions to maintain the ion balance in a cell [27,28]. In *N. sibirica.*, V-H^+^-PPase is encoded by the *NsVP1* gene, and previous studies have shown that *NsVP1* gene expression and enzyme activity increased under salt stress [25], potentially explaining its superior salt tolerance. Thus, another objective of this study was to elucidate whether NsVP1 is a downstream target protein of Ns14-3-3.

## 2. Results

### 2.1. Genome-Wide Identification of Ns14-3-3 Gene Family Members and Characterization of Their Proteins

In this study, we identified nine Ns14-3-3 family members from the N. sibirica genome by bioinformatics methods (Table 1). The lengths of the 14-3-3 genes in N. sibirica ranged from 750 to 798 bp, the numbers of encoded amino acids ranged from 249 (Ns14-3-3 9a) to 265 (Ns14-3-3 1b), and the molecular weights (MWs) ranged from 28.23 kD (Ns14-3-3 9a) to 29.93 kD (Ns14-3-3 1b). The results indicate that the Ns14-3-3 proteins are acidic, with isoelectric points (pIs) less than 7.0. Additionally, the grand average of hydropathy (GRAVY) of the Ns14-3-3 proteins is negative, indicating that they are hydrophilic. The instability coefficients of Ns14-3-3 proteins ranged from 38.01 (Ns14-3-3 1c) to 51.41 (Ns14-3-3 1a), and all of them except Ns14-3-3 1c and Ns14-3-3 5a had high instability indices (>40), indicating that 78% of the Ns14-3-3 proteins were unstable at the theoretical level. Prediction of the possible subcellular localization of the Ns14-3-3 proteins revealed that they are widely distributed in cells, with distributions at the plasma membrane, chloroplast, nucleus, and cytoplasm, with the largest number of Ns14-3-3 proteins being in the nucleus.

### 2.2. Phylogenetic Analyses of 14-3-3 Gene Family Members

**Figure 1:**
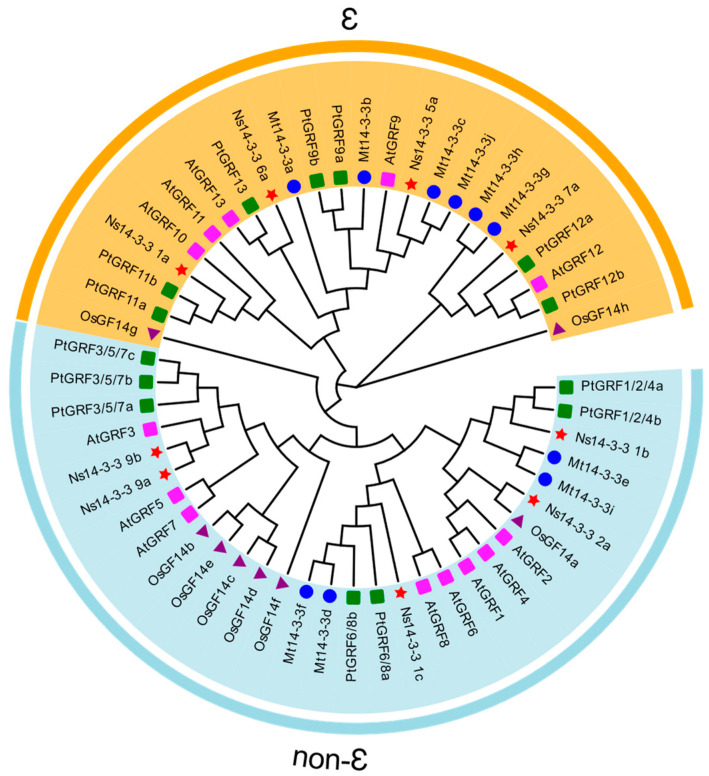
Phylogenetic analysis of *14-3-3* genes in *N. sibirica*, *Arabidopsis thaliana*, *Oryza sativa*, *Medicago truncatula*, and *Populus trichocarpa*. The phylogenetic tree depicts the relationships among 9 *Ns14-3-3* genes (red five-pointed stars), 8 *OsGF14* (purple triangle), 13 *AtGRF* (pink square), 10 *Mt14-3-3* (blue circle), and 14 *PtGRF* (green square) genes. The unrooted tree was constructed using the JTT+G model of MEGA 11.0 and divided into two subfamilies. Different groups are marked with different colored branches, light yellow indicates the *14-3-3* genotype of the ε group and cyan grey indicates the *14-3-3* genotype of the non-ε group.

To investigate the evolutionary relationship of Ns14-3-3 proteins, the *A. thaliana 14-3-3* (*AtGRF*), *Oryza sativa 14-3-3* (*OsGF*), *Medicago truncatula 14-3-3* (*Mt 14-3-3*), and *Populus trichocarpa 14-3-3* (*PtGRF*) genes were selected to construct a phylogenetic tree together with *Ns14-3-3s* (Figure 1). The *Ns14-3-3* gene family can be classified into two types, the ε group and non-ε group, similar to the *14-3-3* gene family of *Arabidopsis thaliana* and three other species. Figure 1 shows that the *Ns14-3-3* genes are only weakly related to the other four species’ *14-3-3* genes. The *Ns14-3-3 2a* gene is in the same minimal sub-branch as *OsGF14a*, while the remaining *Ns14-3-3* genes are in a minimal sub-branch by themselves. Overall, among the four species’ *14-3-3* genes, the *Ns14-3-3* gene is the closest relative to the *Populus trichocarpa 14-3-3* gene. This is likely due to the fact that both are woody plants. The number of ε group members (four) in *Ns14-3-3s* is less than and the number of non-ε group members (five). However, the number of members in the ε and non-ε groups varied among the five species. Some species had more members in the ε group than others, while some had fewer members in the ε group. Additionally, *Populus trichocarpa 14-3-3* genes had an equal number of members in both the ε and non-ε groups, reflecting interspecies differences.

### 2.3. The Multiple Sequence Alignment of Ns14-3-3 Proteins

**Figure 2:**
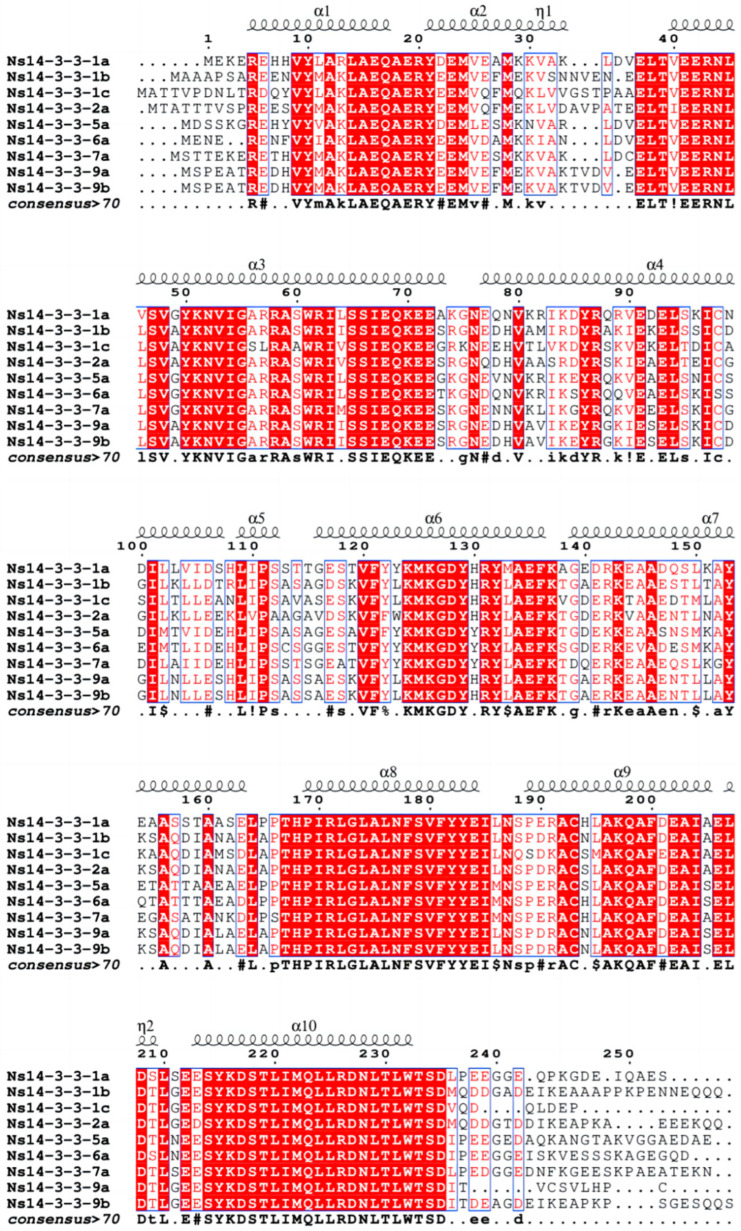
Multiple sequence alignment of all 14-3-3 proteins in *N. sibirica*. The red areas are fully conservative. Ten α-helices were marked as α1–α10. Two 310 helix were marked as η1–η2.

The multiple sequence alignment of the Ns14-3-3 protein family members via ESPript 3.0 software showed a high degree of similarity amongst them (Figure 2). Their identity is 76.99%, the positions of the intermediate regions are highly similar, and they contain the conserved structural domains of the *14-3-3* gene family. Although the structures of the proteins in this family are highly conserved, the N-terminus and C-terminus, which form the core structures responsible for 14-3-3 protein functions, varied considerably according to multiple sequence alignment.

### 2.4. Chromosome Distribution and Synteny Analysis within Species of 14-3-3 Gene Family Members in N. sibirica

**Figure 3:**
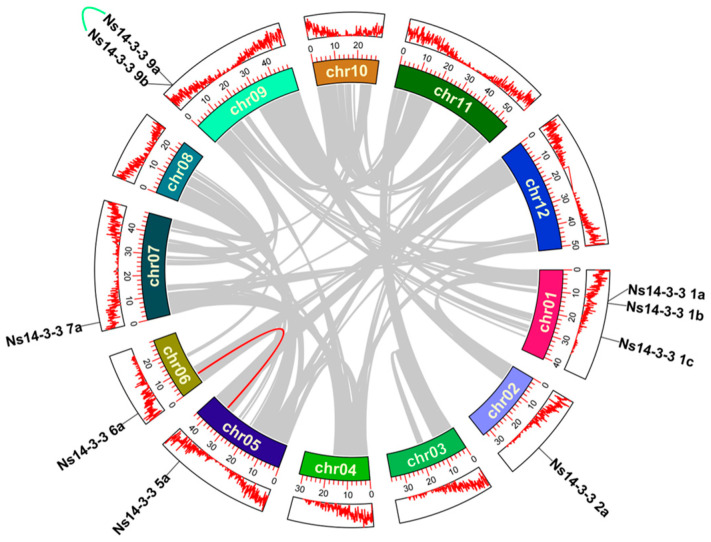
Genomic distribution and duplicate pairs of *Ns14-3-3* genes across the nine *N. sibirica* chromosomes. The first circle displays the names of genes and their corresponding chromosome densities. The second circle is an ideogram of chromosomes, displaying their names and scales indicating their coordinate positions. The *Ns14-3-3* gene is represented by green arcs for tandem repeat pairs and red arcs for fragment repeat pairs.

The positions of the *14-3-3* gene family members in the *N. sibirica* genome were analyzed. The results showed that nine *Ns14-3-3* genes were heterogeneously distributed on six chromosomes (Figure 3). They were most densely distributed on chromosome 01, with three *Ns14-3-3* genes, while there were two genes on chromosome 09 and one on each of the other four chromosomes. Analysis of intragenomic duplication events for all *Ns14-3-3* genes revealed a pair of tandem duplicated genes (*Ns14-3-3 9a* and *Ns14-3-3 9b*) and a pair of genes that underwent fragment duplication events (*Ns14-3-3 5a* and *Ns14-3-3 6a*). This suggests that both tandem and fragment replications contribute to the amplification of the *Ns14-3-3* family, but their numbers are relatively low compared to other species.

### 2.5. Gene Structure and Motif Analysis

**Figure 4:**
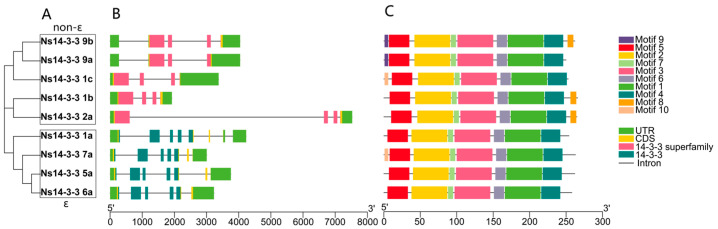
Phylogeny, gene structure, conserved domains, and motifs of *Ns14-3-3* genes. (**A**) Construction of an unrooted neighbor-joining phylogenetic tree comprising nine *Ns14-3-3* gene sequences. (**B**) Structure of exons, introns, conserved domains, and untranslated regions (UTR) in *Ns14-3-3* genes. The green box represents the UTR region in the gene structure, the yellow box represents the CDS region in the gene structure, and the thin gray line represents the intron region in the gene structure. The pink boxes indicate that they contain 14-3-3 superfamily conserved domains and blue boxes indicate that they contain 14-3-3 conserved domains. (**C**) Distribution of conserved motifs within the *14-3-3* gene sequences. The differently colored boxes represent different bases.

To systematically analyze the gene structures of *Ns14-3-3* genes, a *14-3-3* gene structure map (Figure 4) was constructed. Referring to the phylogenetic analysis of *Arabidopsis*, two distinctive groups across three primary branches were identified (Figure 4A). The first two branches form the ε group, with the remaining branch being the non-ε group. The exon/intron maps of the *Ns14-3-3* genes were analyzed, revealing the presence of 3-7 introns. The ε group *Ns14-3-3* gene showed 3-4 introns, while the non-ε group genes presented 5–7 introns (Figure 4B). It is hypothesized that there is an intron loss and an additional exon gain for genes in the *N. sibirica* ε group and non-ε group, and that this exon–intron structural diversity also indicates differences in the amplification and evolution of the genes in the different groups. Conservative domain analysis revealed that the *Ns14-3-3* gene subgroup consists of two protein domains: the ε group, which includes the 14-3-3 domain, and the non-ε group, which includes the 14-3-3 superfamily domain. These findings suggest that there may be functional differences between the ε and non-ε groups. Our analysis of conserved motifs revealed that the motif composition among all subgroup members of *Ns14-3-3* genes was relatively uniform. A total of 10 conserved motifs of *Ns14-3-3* gene family proteins were predicted by MEME, among which motifs 1 to 7 were conserved in ε group and non-ε group *Ns14-3-3* genes and were the characteristic motifs of *Ns14-3-3s*, suggesting that they may be necessary for protein composition. Notably, motifs 8 and 9 are specific to non-ε groups, which may explain the functional differences between ε and non-ε groups.

### 2.6. Analysis of the Cis-Regulatory Elements of Ns14-3-3 Gene Family Member Promoters

**Figure 5:**
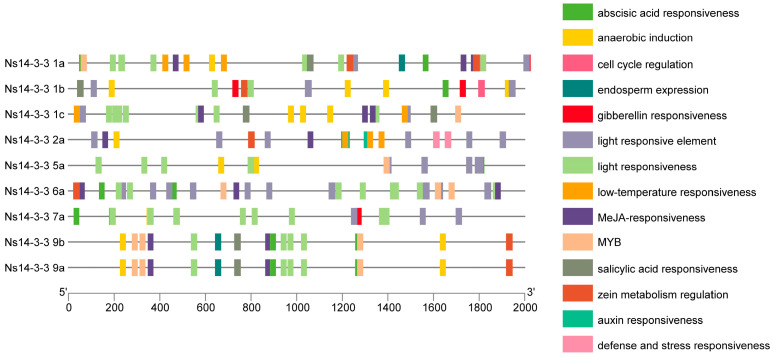
Visualization of the results of an analysis of cis-regulatory elements within the promoters of *Ns14-3-3* gene family members.

To comprehend the expression and regulatory traits of the *Ns14-3-3* gene family, we analyzed the promoter sequence of each gene member. This was performed by examining the 2 kb region upstream of the coding region using the PlantCARE database (Figure 5). The promoter sequence of *Ns14-3-3s* contains cis-acting elements that respond to light, abiotic stress, external stresses, phytohormones, and plant growth and development. *Ns14-3-3s* contains the largest number of cis-acting elements involved in light response. The cis-acting elements that are responsive to plant hormones are diverse, including abscisic acid, gibberellin, auxin, methyl jasmonate, and salicylic acid. In addition, cis-acting regulatory elements involved in plant stress response also account for a certain proportion, such as the cis-acting regulatory elements essential for anaerobic induction and low-temperature responsiveness. This indicates that *Ns14-3-3s* may be regulated by a hormone-induced variety and stress signals, and are involved in various physiological, metabolic processes and stress response pathways in plants. Notably, cis-acting elements regulating the cell cycle were only found in *Ns14-3-3 1a* and *Ns14-3-3 1b*. *Ns14-3-3 1a* contained a cis-acting element that regulates endosperm expression, and *Ns14-3-3 1b* contained a cis-acting element involved in palisade mesophyll cell differentiation. This suggests that *Ns14-3-3 1a* and *Ns14-3-3 1b* may play important roles in the regulation of *N. sibirica* growth and development, and they may be involved in plant-specific growth and development processes. Furthermore, the cis-acting element involved in defense and stress responses was exclusively identified in *Ns14-3-3 2a*, which indicated that *Ns14-3-3 2a* has a potential role in enhancing plant resilience to adverse conditions.

### 2.7. Transcription Profiling of Ns14-3-3 Genes

**Figure 6:**
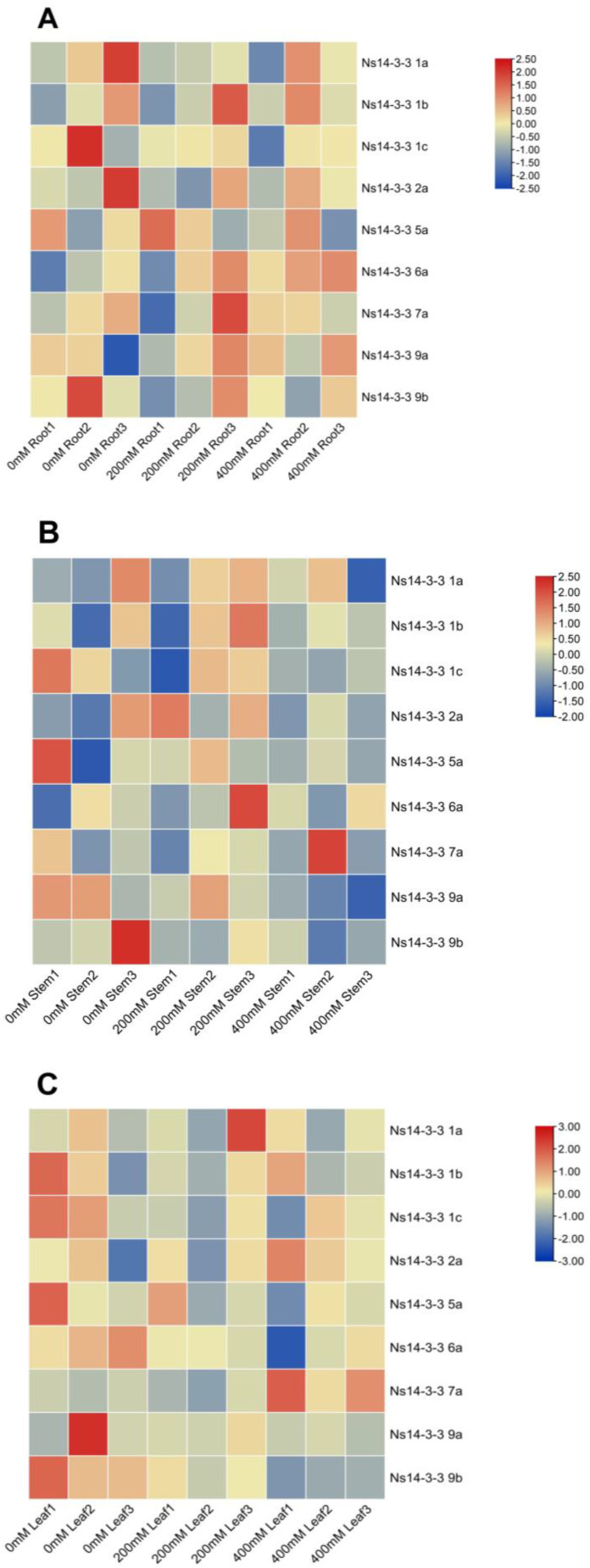
Transcriptome analysis of *Ns14-3-3s* in roots (**A**), stems (**B**), and leaves (**C**) under salt stress. The figure displays the concentration of salt treatment on the lower part, with the gene name located on the right. The gradient from blue to red represents low to high expression.

The analysis of cis-acting elements suggests that *Ns14-3-3s* may play a role in responding to abiotic stress. Given the prominent role of *Ns14-3-3s* in salt tolerance, we analyzed the transcriptomes of *Ns14-3-3s* in roots (Figure 6A), stems (Figure 6B), and leaves (Figure 6C) separately under salt stress. The expression of *Ns14-3-3s* in different tissues varied with changes in salt concentration. The four types can be roughly divided based on the expression of *Ns14-3-3s* with varying salt concentrations: increased expression, decreased expression, initially increased then decreased expression, and initially decreased then increased expression. The transcriptome analysis revealed that as the concentration of salt treatment increased, the expression level of *Ns14-3 1a* in the roots decreased, while the expression levels of *Ns14-3-3 6a* and *Ns14-3-3 9a* increased. The expression of *Ns14-3-3 9a* and *Ns14-3-3 9b* in stems decreased with increasing salt treatment concentration. The expression of *Ns14-3-3 5a*, *Ns14-3-3 6a*, and *Ns14-3-3 9b* in leaves decreased, while the expression of *Ns14-3-3 2a* and *Ns14-3-3 7a* increased with increasing salt concentration. The results suggest that *Ns14-3-3s* responds to salt stress, but further verification is needed to determine which specific members respond and how they respond.

### 2.8. Expression Profiles of Ns14-3-3 Genes in Various Tissues of N. sibirica

**Figure 7:**
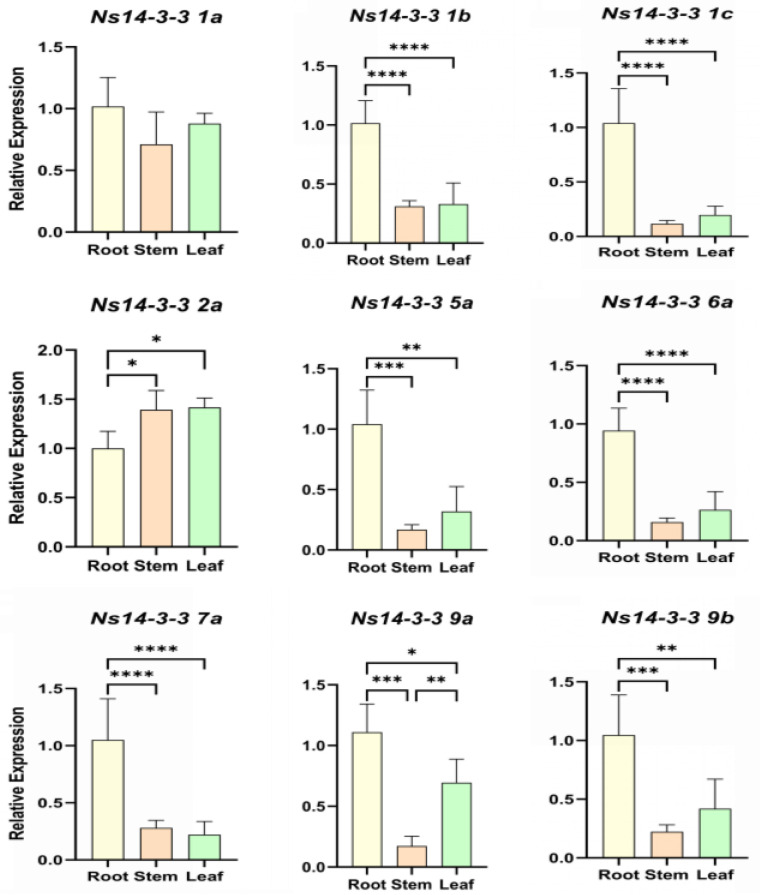
Gene expression of *Ns14-3-3s* in roots, stems and leaves under normal conditions. In the figure, the horizontal axis is three different tissues of roots (yellow), stems (orange) and leaves (green), and the vertical axis is the relative expression of each *Ns14-3-3* gene in roots, stems and leaves (with roots as the standard one). The mean standard deviation (SD) of three independent biological replicates were presented, with error bars representing the SD. Different number of asterisks indicate significantly different values (* *p* < 0.05, ** *p* < 0.01, *** *p* < 0.001, and **** *p* < 0.0001).

To confirm the response of *Ns14-3-3s* members in salt tolerance, we used qRT-PCR to assess the expression pattern of the *Ns14-3-3* genes in different tissues (Figure 7). However, there were differences in the expression patterns and levels of different genes. Overall, *Ns14-3-3s* expression was highest in roots, followed by leaves, and lowest in stems. All *Ns14-3-3* genes, except for *Ns14-3-3 2a*, exhibited the highest expression levels in roots. *Ns14-3-3 2a* was expressed at the highest level in stems, while there was little variation in expression among the three tissues of roots, stems, and leaves. In addition, *Ns14-3-3 1a* was highly expressed in stems. The expression levels of *Ns14-3-3 1a*, *Ns14-3-3 2a*, and *Ns14-3-3 9a* were high but not the highest in leaves.

### 2.9. Expression Patterns of Ns14-3-3s under Salt Stress

**Figure 8:**
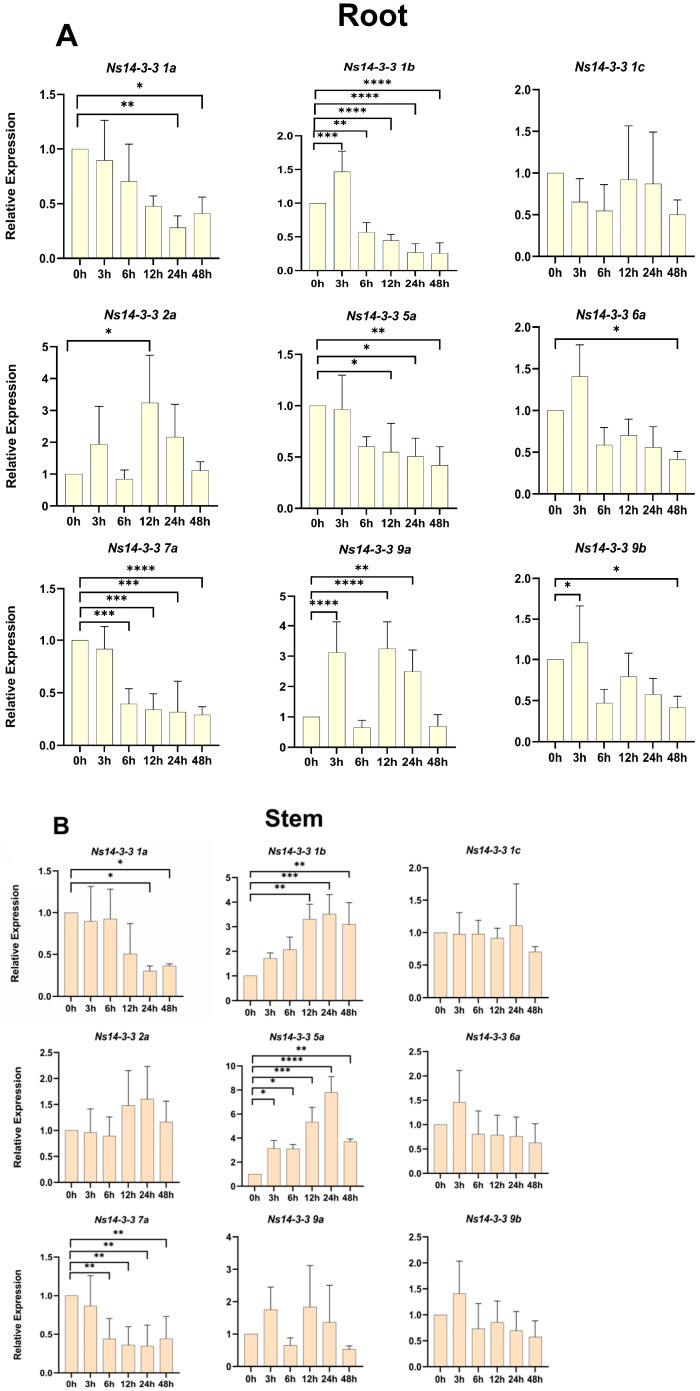

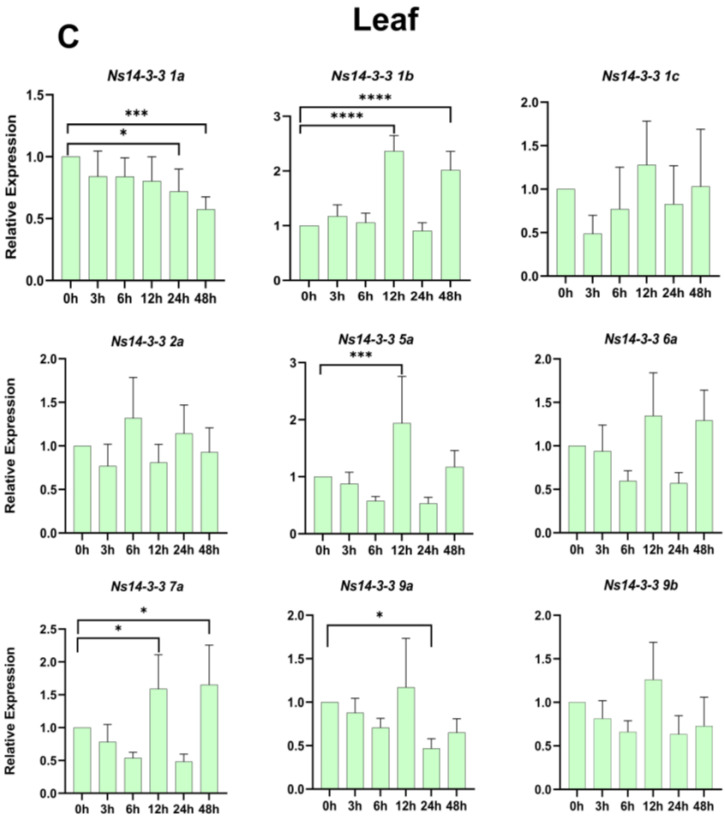
The gene expression of *Ns14-3-3s* in roots (**A**), stems (**B**), and leaves (**C**) changes with variations in salt treatment time. In the figure, the abscissa is six different treatment periods under 200 mM NaCl treatment, and the ordinate is the relative expression of each *Ns14-3-3* gene. The mean standard deviation (SD) of three independent biological replicates were presented, with error bars representing the SD. Different number of asterisks indicate significantly different values (* *p* < 0.05, ** *p* < 0.01, *** *p* < 0.001, and **** *p* < 0.0001).

The expression levels of some members of the *Ns14-3-3s* appeared to be induced under salt stress, and the expression patterns in roots (Figure 8A), stems (Figure 8B), and leaves (Figure 8C) were not identical. In roots, there was a significant decrease in expression of *Ns14-3-3 1b* (a decrease of 43.3% to 74.4%), *Ns14-3-3 5a* (a decrease of 47.1% to 57.9%), and *Ns14-3-3 7a* (a decrease of 60.5% to 71%) with increasing salt treatment time. *Ns14-3-3 1a*, *Ns14-3-3 6a*, and *Ns14-3-3 9b* showed a tendency to decrease in their expression with increasing salt treatment time. *Ns14-3-3 2a* and *Ns14-3-3 9a* exhibited an oscillatory pattern of increase and decrease with increasing salt treatment time. In the stems, *Ns14-3-3 1b* (an increase between 209 and 251%) and *Ns14-3-3 5a* (an increase between 212 and 680%) exhibited a significant increase in expression as the duration of salt treatment increased. With increasing salt treatment time, the expression of *Ns14-3-3 7a* significantly decreased (between 56 and 65%), *Ns14-3-3 1a* exhibited a decreasing trend. In the leaves, *Ns14-3-3 1a* presented a time-dependent decreasing trend. *Ns14-3-3 1b*, *Ns14-3-3 5a,* and *Ns14-3-3 7a* exhibited an oscillatory pattern of increase and decrease with increasing salt treatment time. It is noteworthy that they all showed a significant rise at the time point of 12 h salt treatment (between 59 and 136%), which seems to be a critical point for them to respond to salt stress. In addition, a response to salt stress was observed in all tissues of *Ns14-3-3 1a*, *Ns14-3-3 1b*, *Ns14-3-3 5a,* and *Ns14-3-3 7a*.

### 2.10. Validation of Interactions between Ns14-3-3s and NsVP1

**Figure 9:**
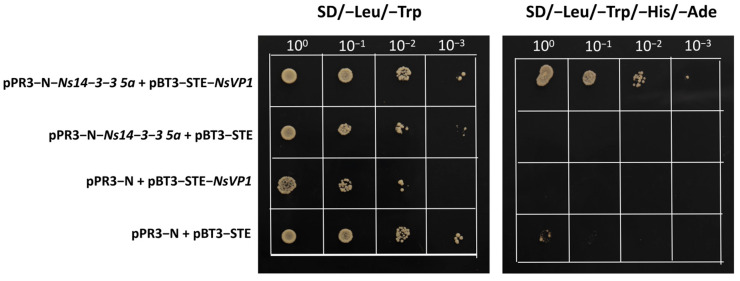
Yeast two-hybrid assays of interactions between candidate Ns14-3-3 proteins and NsVP1.

The transcript levels of *Ns14-3-3 1a*, *Ns14-3-3 1b*, *Ns14-3-3 5a*, and *Ns14-3-3 7a* all showed salt-induced expression. These genes may play a crucial role in salt tolerance and response to salt stress. To further understand the role of these genes in salt tolerance, we tried to find their downstream target genes. Specifically, we have verified their interactions with NsVP1 using the yeast two-hybrid system (Figure 9). All four combinations were able to grow on SD/-Leu/-Trp medium. PPR3-N+pBT3-STE was used as a negative control and did not grow on SD/-Trp/-Leu/-His/-Ade medium. PPR3-N-*Ns14-3-3 5a*+pBT3-STE and pPR3-N+pBT3-STE-*NsVP1* did not grow on SD/-Trp/-Leu/-His/-Ade medium, indicating that auto-activation is not present. PPR3-N-*Ns14-3-3 5a*+pBT3-STE-*NsVP1* was grown on SD/-Trp/-Leu/-His/-Ade medium, indicating a reciprocal relationship between Ns14-3-3 5a and NsVP1.

## 3. Discussion

Many studies have shown that 14-3-3 proteins play important roles in a variety of biological processes and are key regulators of various signal transduction pathways in plants. However, there are limited studies on 14-3-3 proteins in halophytes, and their function need to be explored in more detail. In this study, we used bioinformatics methods to identify nine members of the *14-3-3* gene family in *N. sibirica*. Regarding physicochemical properties, it was found that Ns14-3-3 proteins are acidic and hydrophilic, with most being unstable and having a protein molecular weight of around 29 kDa. These characteristics are consistent with those of 14-3-3 proteins and previous studies on woody plants [21,29]. Multiple sequence comparisons of Ns14-3-3 proteins indicate that their protein structures are conserved, but there are variations in their N-terminal and C-terminal sequences and structures. The 14-3-3 protein’s N-terminus and C-terminus are essential for its function. The functional site for membrane binding is located at the N-terminus, whereas the site for interaction with the target protein is located at the C-terminus [2]. The variations in sequence and structure of the N-terminus and C-terminus of the *Ns14-3-3s* proteins indicate that they have distinct functions.

Similar to other species such as *Arabidopsis* [19], tomato [21], and mango [22], all nine members of the *Ns14-3-3* gene family are divided into two main categories based on phylogenetic relationships, ε and non-ε. The ε group and non-ε group display different structural characteristics in their exons and introns. The ε group has a higher number of introns and is more conserved than the non-ε group. The ε group consists of 14-3-3 conserved domains, while the non-ε group consists of 14-3-3 superfamily conserved domains. The distribution of the *Ns14-3-3* gene family on chromosomes is non-random and uneven. Some chromosomes have multiple *Ns14-3-3* genes, chr01 contains three *Ns14-3-3* genes, and chr09 has two. The presence of multiple *Ns14-3-3* genes on a single chromosome is widespread and has been found in various plants, including *Arabidopsis* [19], soybean [30], and *M. truncatula* [31].

The main drivers of gene family amplification are segmental duplications, tandem duplications, and transposition events [32]. Segmental duplication plays an important role in the amplification of *14-3-3* gene family and is the main mechanism leading to the increase in the *14-3-3* gene family, which has been reported in soybean [30] and tomato [21]. In *N. sibirica*, we identified a tandem duplication gene pair (*Ns14-3-3 9a* and *Ns14-3-3 9b*) and a fragment duplication-based pair (*Ns14-3-3 5a* and *Ns14-3-3 6a*). These findings suggested that both tandem and fragment duplications contribute to the amplification of the *Ns14-3-3* family. However, there is a relatively low number of fragment replication-based pairs in *N. sibirica* compared to other species, which may be attributed to the high conservation of *Ns14-3-3s*.

Cis-acting elements are crucial in identifying genes involved in specific functions related to plant growth, development, and stress responses [33]. A previous study discovered that overexpression of *14-3-3* (*SiGRF1*) from foxtail millet in *Arabidopsis* stimulates flowering under salt stress [34]. The researchers hypothesized that *SiGRF1* could enhance salt tolerance by accelerating the plant’s life cycle, thus reducing exposure to salt stress [34]. Perhaps the *Ns14-3-3 1a* and *Ns14-3-3 1b* genes play similar roles. Furthermore, the promoter of the *Ns14-3-3* gene contains several cis-acting elements that respond to various phytohormone, including auxin, salicylic acid (SA), methyI jasmonate (JA), abscisic acid (ABA), and gibberellin (GA). This suggests that *Ns14-3-3s* can be induced by these hormones. The number of cis-acting elements in response to ABA is the largest, and the number of JA is the second largest. It was found in barley [35], wheat [36], and common bean [13] that 14-3-3 protein was induced by an ABA signal and involved in ABA signal regulation. ABA is a crucial cellular signal that regulates the expression of salt-responsive genes at high concentrations [37]. ABA and JA also enhance the activity of antioxidant enzymes, thereby improving plant salt tolerance [38,39,40]. The expression of the *Pp14-3-3a* in pear fruit is suppressed by salicylic acid [41]. Salicylic acid can regulate carbohydrate metabolism to reduce the negative effects of salt stress in chickpeas [42]. In tobacco, 14-3-3 protein negatively regulates GA expression by interacting with *REPRESSION OF SHOOT GROWTH* (RSG) [43]. During the later stages of salt stress, the concentration of GA decreases, leading to an increase in the number of lateral roots as a response to the stress [44,45]. The above results suggest that *Ns14-3-3s* may play an important role in the response to salt stress in *N. sibirica*. It is important to note that the presence of cis-acting elements involved in defense, stress responsiveness, and auxin response is limited to *Ns14-3-3 2a*. This implies that *Ns14-3-3 2a* plays a significant role in stress tolerance, including contributing to salinity resistance.

The expression patterns of *Ns14-3-3s* varied across different tissues and were also affected by different salt treatments. The majority of *Ns14-3-3s* members were expressed most abundantly in roots, which is consistent with the previously reported expression patterns of several 14-3-3 family members. The expressions of *Ns14-3-3 1a* in roots, stems and leaves were similar. *Ns14-3-3 1a*, *Ns14-3-3 1b*, *Ns14-3-3 5a,* and *Ns14-3-3 7a* exhibited sensitivity to salt. With the increase in salt stress time, *Ns14-3-3 1a* showed a decreasing trend in all three tissues, which may negatively regulate salt tolerance. The expression levels of *Ns14-3-3 1b* and *Ns14-3-3 5a* decreased in roots but increased in stems and leaves as salt stress time increased. Additionally, the expression of *Ns14-3-3 7a* decreased in roots and stems but increased in leaves. This suggests that *Ns14-3-3s* genes respond to salt stress by regulating expression with tissues specificity. To further understand the role of these genes in salt tolerance, we tried to find their downstream target genes. It was found that Ns14-3-3 5a interacted with NsVP1 through yeast two-hybrid experiments. Plants can maintain a higher cytoplasmic K^+^/Na^+^ ratio and have greater tissue salt tolerance by translocating salts into vacuole for its sequestration in a process mediated by Na^+^/H^+^ antiporter encoded by *NHX* genes [46,47,48]. NsVP1 pumps H^+^ from the cytoplasm to the vacuolar by hydrolyzing pyrophosphate, creating a trans-vesicular membrane proton gradient for Na^+^ translocation by NHX [49,50]. Previous studies on *N.sibirica* have found that the tissue salt tolerance of leaves is its main salt tolerance strategy [25]. Under salt stress, the expression of *NsVP1* and *NsNHX1* genes is up-regulated and the activities of vacuolar H^+^-ATPase and H^+^-PPase enzymes are increased, resulting in an increase in ions compartmentation in the vacuolar. The expression of Ns14-3-3 5a, which interacts with NsVP1, decreases in roots and increases in stems and leaves. This implies that Ns14-3-3 5a may respond to salt stress by enhancing the enzymatic and proton pumping activities of NsVP1 through interactions with NsVP1, leading to a decrease in vacuolar ions sequestration in the root and an increase in ions compartmentation in the stems and leaves. Being a multibranched shrub, *N. sibirica* can compartmentalize more salt ions (Na^+^ and Cl^−^) in stems and leaves, explaining its superior salt tolerance as compared with other plants.

## 4. Materials and Methods

### 4.1. Identification and Sequence Analysis of 14-3-3 Gene Family Members in N. sibirica

To identify potential members of the *14-3-3* gene family in the *N. sibirica* genome, the *N. sibirica* genome (unpublished) was used. Sequences of 13 members of the *Arabidopsis 14-3-3* family were obtained from the TIAR database [51] (http://www.arabidopsis.org/, accessed on 8 September 2023) as query sequences and local BLAST searches for sequences of *14-3-3* homologous genes in the *N. sibirica* genome database were performed using TBtools [52]. The screened Ns14-3-3 proteins were self-blasted by NCBI database (https://www.ncbi.nlm.nih.gov/, accessed on 8 September 2023) and Visualize NCBI CDD Domian Pattern was used to identify the structural domain of the retrieved Ns14-3-3 protein sequence, and then the proteins with missing structural domains and repetitive proteins were removed. Finally, the candidate genes of the *Ns14-3-3* family were obtained. The ExPAsy ProtParam tool (https://web.expasy.org/protparam/, accessed on 15 September 2023) was used to predict several parameters of each Ns14-3-3 protein, such as length, amino acid count, molecular weight, isoelectric point, instability index, and grand average of hydropathicity. The subcellular localization of each member was predicted using the subcellular localization prediction tool WoLF PSORT (https://wolfpsort.hgc.jp, accessed on 16 September 2023).

### 4.2. Chromosomal Location and Gene Structure Analysis

The chromosomal locations of the *Ns14-3-3* gene family members were extracted from the gff3 file of the *N. sibirica* genome annotation, and a map of the chromosomal gene distribution was constructed with TBtools [52]. The amino acid sequences of the proteins were analyzed using the MEME software (http://meme-suite.org, accessed on 30 September 2023). The number of motifs was set to 10, and the other parameters were set to their default values [53]. Domain analysis was performed using NCBI Batch CD-search (https://www.ncbi.nlm.nih.gov/Structure/bwrpsb/bwrpsb.cgi, accessed on 30 September 2023). Exon and intron numbers were predicted using TBtools v2.067 software Gene Structure Shower. Finally, the above data was visualized using TBtools [52].

### 4.3. Sequence Alignment, Phylogenetic Analysis, and Synteny Analysis of Ns14-3-3s

The protein sequence conservation of amino acids was analyzed using MEGA 11 software, and its visualization was created using ESPript 3.0 (https://espript.ibcp.fr/ESPript/ESPript/index.php, accessed on 8 October 2023). The amino acid sequences of 14-3-3 proteins from *Arabidopsis*, *Oryza sativa*, *Medicago truncatula*, and *Populus trichocarpa* were obtained from the phytozome 13 genome databases (https://phytozome-next.jgi.doe.gov/, accessed on 8 October 2023). A phylogenetic tree was then constructed using the maximum likelihood method in MEGA 11.0 (bootstrap set to 1000) [54].

### 4.4. Analysis of Cis-Acting Elements in the Promoter Region of Ns14-3-3s

Sequences in the 2000 base pairs region upstream of the start codon of each *Ns14-3-3* gene were extracted from the *N. sibirica* genome-annotated gff3 file. Cis-regulatory elements within the promoter were predicted using PlantCARE [55] (http://bioinformatics.psb.ugent.be/webtools/plantcare/html, accessed on 19 October 2023) and visualized with Tbtools [52].

### 4.5. Transcriptional Profiling of Ns14-3-3s

Transcriptome data of *N. sibirica* treated in 0 mmol/L, 200 mmol/L, and 400 mmol/L NaCl solution (SRX9833449) were downloaded from the NCBI database. The transcriptome data in different tissues of roots, stems, and leaves under salt stress were obtained by collating, and the heat map of expression was drawn using HeatMap function of TBtools [52].

### 4.6. Plant Material and Treatments

Seeds of *N. sibirica* were collected from Keluke beach saline-alkaline soil in the Qaidam Basin of Qinghai Province, China (37°10′–37°20′ N, 96°49′–97°37′ E). Following seed germination, aseptic propagation was conducted through histoculture. Well-grown and uniform plants were randomly selected for treatment with 0, 200 mmol/L NaCl after 50 days. Tissue samples from the roots, stems, and leaves of the histocultured seedlings were collected after treatments of 0, 3, 6, 12, 24, and 48 h. The samples were washed with purified water, immediately frozen in liquid nitrogen, and stored at −80 °C for RNA extraction. The aforementioned procedures were conducted in three biological replicates [25].

### 4.7. Total RNA Extraction, Quantitative Real-Time PCR (qRT-PCR), and Statistical Analysis

RNA extraction was performed using the E.Z.N.A.^®^ Plant Kit (OMEGA Bio-Tek, Doraville, GA, USA) according to the product manual. According to the HiFiScript gDNA Removal RT MasterMix (CoWin Biosciences, Beijing, China) kit instructions, 1 μg of high-quality RNA extracted from each sample was reverse transcribed into first-strand cDNA, the obtained cDNA was then used for qRT-PCR. Primer Premier 5.0 software was used to design qRT-PCR primers, and ACT7 was selected as the internal reference gene [56], all primers were synthesized by RuiBiotech company (Beijing, China). cDNA was amplified by using UItraSYBR Mixture (CoWin Biosciences, Beijing, China), qRT-PCR was performed in 384-well plates by using a LightCycler^®^ 480II Real-Time PCR System (Roche Molecular Systems, Mannheim, Germany). The amplification conditions were as follows: an initial step of 95 °C for 10 min, followed by 40 cycles of 95 °C for 10 s, 55 °C for 30 s and 72 °C for 32 s. All qRT-PCR assays were performed three times as independent biological replicates, and the mean of the cycle threshold (C_t_) was obtained. Relative gene expression levels were calculated using the 2^−∆∆Ct^ method [57]. Three independent biological replicates of each test were averaged and then plotted using GraphPad Prism 9.5. The primers used in this assay are listed in Supplementary Materials file Table S1.

### 4.8. Yeast Two-Hybrid Experiment

The coding sequences for *Ns14-3-3 1a*, *Ns14-3-3 1b*, *Ns14-3-3 5a*, and *Ns14-3-3 7a* were cloned into the prey vector pBT3-STE, and the coding sequence for *NsVP1* was cloned into the bait vector pPR3-N, and then transformed into the yeast strain NMY51 according to the manual [58]. The yeast was transformed according to the instructions of the Classic Yeast Transformation Kit (Coolaber, Beijing, China). The interactions between bait and prey were then tested on SD medium without Leu, Trp, His, and Ade [58]. The primers used for yeast expression vector construction were listed in Supplementary Materials file Table S2.

## 5. Conclusions

In summary, we identified nine *14-3-3* genes from the *N. sibirica* genome. Consistent with previous studies, these genes were divided into two groups, the ε group and the non-ε group. The evolutionary analyses indicate that these genes have undergone both segmental and tandem duplications during evolution. The analysis of cis-acting elements indicates their involvement in plant growth and development, plant hormone response, and abiotic stress tolerance. Transcriptome and gene expression analysis revealed that *Ns14-3-3s* played a crucial role in the response of *N. sibirica* to salt stress. It is likely that *Ns14-3-3 1a*, *Ns14-3-3 1b*, *Ns14-3-3 5a,* and *Ns14-3-3 7a* are candidate genes involved in the salt stress response. The results of the yeast two-hybrid assay showed that Ns14-3-3 5a interacts with NsVP1, which may increase ion compartmentalization in stems and leaves by enhancing the enzyme activity and proton pumping activity of NsVP1, thereby improving plant salt tolerance. The results indicate that *Ns14-3-3s* are involved in the response to salt stress and can regulate plant salt tolerance through interactions with proton pump. Overall, our study provided new clues for discovering important candidate genes for salt tolerance in *N. sibirica*. Further research is needed to establish the role of *Ns14-3-3s* in regulatory networks in *N. sibirica* and its impact on ionic homeostasis and regulation of key transporters involved in this process.

## Figures and Tables

**Table 1:** Information concerning *14-3-3* genes in *N. sibirica*.

Gene Name	ID	Chr	aa	pI	II	MW (Da)	GRAVY	Subcellular Localization
*Ns14−3−3 1a*	Nisib01aG0106800.1	Chr01	253	4.96	51.41	28,887.41	−0.626	plas
*Ns14−3−3 1b*	Nisib01aG0110100.1	Chr01	265	4.73	44.81	29,929.55	−0.563	plas
*Ns14−3−3 1c*	Nisib01aG0151400.1	Chr01	252	4.76	38.01	28,539.45	−0.287	chlo
*Ns14−3−3 2a*	Nisib02aG0114500.1	Chr02	264	4.72	49.62	29,789.47	−0.492	chlo
*Ns14−3−3 5a*	Nisib05aG0198000.1	Chr05	261	4.78	38.18	29,371.89	−0.55	nucl
*Ns14−3−3 6a*	Nisib06aG0046300.1	Chr06	257	4.73	44.24	29,290.77	−0.615	nucl
*Ns14−3−3 7a*	Nisib07aG0034600.1	Chr07	262	4.90	48.29	29,860.49	−0.702	nucl
*Ns14−3−3 9a*	Nisib09aG0041400.2	Chr09	249	4.97	45.09	28,234.04	−0.310	cyto
*Ns14−3−3 9b*	Nisib09aG0041400.1	Chr09	261	4.78	49.08	29,479.14	−0.47	nucl

Abbreviations: Chr = chromosome position, aa = amino acid number, pI = Isoelectric point, II = instability index, MW = molecular weight, plas = plasma membrane, chlo = chloroplast, nucl = nucleus, cyto = cytoplasm.

## Data Availability

All datasets presented in this study are included in the article/Supplementary Table.

## Supplementary Materials

The following supporting information can be downloaded at: https://www.mdpi.com/article/10.3390/ijms25063432/s1.

## References

[1] Liu Q., Zhang S., Liu B. (2016). 14-3-3 Proteins: Macro-Regulators with Great Potential for Improving Abiotic Stress Tolerance in Plants. Biochem. Biophys. Res. Commun..

[2] Zhao X., Li F., Li K. (2021). The 14-3-3 Proteins: Regulators of Plant Metabolism and Stress Responses. Plant Biol..

[3] Stevers L.M., Sijbesma E., Botta M., MacKintosh C., Obsil T., Landrieu I., Cau Y., Wilson A.J., Karawajczyk A., Eickhoff J. (2018). Modulators of 14-3-3 Protein-Protein Interactions. J. Med. Chem..

[4] Aitken A. (2006). 14-3-3 Proteins: A Historic Overview. Semin. Cancer Biol..

[5] Chevalier D., Morris E.R., Walker J.C. (2009). 14-3-3 and FHA Domains Mediate Phosphoprotein Interactions. Annu. Rev. Plant Biol..

[6] Camoni L., Visconti S., Aducci P., Marra M. (2018). 14-3-3 Proteins in Plant Hormone Signaling: Doing Several Things at Once. Front. Plant Sci..

[7] Oecking C., Jaspert N. (2009). Plant 14-3-3 Proteins Catch up with Their Mammalian Orthologs. Curr. Opin. Plant Biol..

[8] Huang Y., Wang W., Yu H., Peng J., Hu Z., Chen L. (2022). The Role of 14-3-3 Proteins in Plant Growth and Response to Abiotic Stress. Plant Cell Rep..

[9] Yao Y., Du Y., Jiang L., Liu J.-Y. (2007). Molecular Analysis and Expression Patterns of the *14-3-3* Gene Family from *Oryza Sativa*. J. Biochem. Mol. Biol..

[10] Wei X., Zhang Z., Li Y., Wang X., Shao S., Chen L., Li X. (2009). Expression Analysis of Two Novel Cotton *14-3-3* Genes in Root Development and in Response to Salt Stress. Prog. Nat. Sci..

[11] Xu W.F., Shi W.M. (2007). Mechanisms of Salt Tolerance in Transgenic *Arabidopsis Thaliana* Constitutively Overexpressing the Tomato 14-3-3 Protein TFT7. Plant Soil.

[12] He Y., Zhang Y., Chen L., Wu C., Luo Q., Zhang F., Wei Q., Li K., Chang J., Yang G. (2017). A Member of the *14-3-3* Gene Family in *Brachypodium Distachyon*, BdGF14d, Confers Salt Tolerance in Transgenic Tobacco Plants. Front. Plant Sci..

[13] Gao G., Li Y., Sun W., He X., Li R., Jin D., Qi X., Liu Z., Bian S. (2018). Functional Roles of Two *14-3-3s* in Response to Salt Stress in Common Bean. Acta Physiol. Plant..

[14] Zhou H., Lin H., Chen S., Becker K., Yang Y., Zhao J., Kudla J., Schumaker K.S., Guo Y. (2014). Inhibition of the *Arabidopsis* Salt Overly Sensitive Pathway by 14-3-3 Proteins. Plant Cell.

[15] Latz A., Becker D., Hekman M., Müller T., Beyhl D., Marten I., Eing C., Fischer A., Dunkel M., Bertl A. (2007). TPK1, a Ca^2+^-Regulated *Arabidopsis* Vacuole Two-Pore K^+^ Channel Is Activated by 14-3-3 Proteins. Plant J. Cell Mol. Biol..

[16] Latz A., Mehlmer N., Zapf S., Mueller T.D., Wurzinger B., Pfister B., Csaszar E., Hedrich R., Teige M., Becker D. (2013). Salt Stress Triggers Phosphorylation of the *Arabidopsis* Vacuolar K^+^ Channel TPK1 by Calcium-Dependent Protein Kinases (CDPKs). Mol. Plant.

[17] Fuglsang A.T., Guo Y., Cuin T.A., Qiu Q., Song C., Kristiansen K.A., Bych K., Schulz A., Shabala S., Schumaker K.S. (2007). *Arabidopsis* Protein Kinase PKS5 Inhibits the Plasma Membrane H^+^ -ATPase by Preventing Interaction with 14-3-3 Protein. Plant Cell.

[18] Gupta A., Shaw B.P., Sahu B.B. (2021). Post-Translational Regulation of the Membrane Transporters Contributing to Salt Tolerance in Plants. Funct. Plant Biol. FPB.

[19] Rosenquist M., Alsterfjord M., Larsson C., Sommarin M. (2001). Data Mining the *Arabidopsis* Genome Reveals Fifteen *14-3-3* Genes. Expression Is Demonstrated for Two out of Five Novel Genes. Plant Physiol..

[20] Yashvardhini N., Bhattacharya S., Chaudhuri S., Sengupta D.N. (2018). Molecular Characterization of the *14-3-3* Gene Family in Rice and Its Expression Studies under Abiotic Stress. Planta.

[21] Jia C., Guo B., Wang B., Li X., Yang T., Li N., Wang J., Yu Q. (2022). Genome-Wide Identification and Expression Analysis of the *14-3-3* (TFT) Gene Family in Tomato, and the Role of *SlTFT4* in Salt Stress. Plants.

[22] Xia L., He X., Huang X., Yu H., Lu T., Xie X., Zeng X., Zhu J., Luo C. (2022). Genome-Wide Identification and Expression Analysis of the *14-3-3* Gene Family in Mango (*Mangifera indica* L.). Int. J. Mol. Sci..

[23] Zhang P., Zhang F., Wu Z., Cahaeraduqin S., Liu W., Yan Y. (2023). Analysis on the Salt Tolerance of *Nitraria Sibirica* Pall. Based on Pacbio Full-Length Transcriptome Sequencing. Plant Cell Rep..

[24] Li H., Tang X., Yang X., Zhang H. (2021). Comprehensive Transcriptome and Metabolome Profiling Reveal Metabolic Mechanisms of *Nitraria Sibirica* Pall. to Salt Stress. Sci. Rep..

[25] Tang X., Zhang H., Shabala S., Li H., Yang X., Zhang H. (2021). Tissue Tolerance Mechanisms Conferring Salinity Tolerance in a Halophytic Perennial Species *Nitraria Sibirica* Pall. Tree Physiol..

[26] Hsu Y.-D., Huang Y.-F., Pan Y.-J., Huang L.-K., Liao Y.-Y., Lin W.-H., Liu T.-Y., Lee C.-H., Pan R.-L. (2018). Regulation of H^+^-Pyrophosphatase by 14-3-3 Proteins from *Arabidopsis Thaliana*. J. Membr. Biol..

[27] Martinoia E., Maeshima M., Neuhaus H.E. (2007). Vacuolar Transporters and Their Essential Role in Plant Metabolism. J. Exp. Bot..

[28] Mansour M.M.F. (2023). Role of Vacuolar Membrane Transport Systems in Plant Salinity Tolerance. J. Plant Growth Regul..

[29] Tian F., Wang T., Xie Y., Zhang J., Hu J. (2015). Genome-Wide Identification, Classification, and Expression Analysis of *14-3-3* Gene Family in *Populus*. PLoS ONE.

[30] Wang Y., Ling L., Jiang Z., Tan W., Liu Z., Wu L., Zhao Y., Xia S., Ma J., Wang G. (2019). Genome-Wide Identification and Expression Analysis of the *14-3-3* Gene Family in Soybean (*Glycine Max*). PeerJ.

[31] Qin C., Cheng L., Shen J., Zhang Y., Cao H., Lu D., Shen C. (2016). Genome-Wide Identification and Expression Analysis of the *14-3-3* Family Genes in *Medicago truncatula*. Front. Plant Sci..

[32] Kong H., Landherr L.L., Frohlich M.W., Leebens-Mack J., Ma H., dePamphilis C.W. (2007). Patterns of Gene Duplication in the Plant SKP1 Gene Family in *Angiosperms*: Evidence for Multiple Mechanisms of Rapid Gene Birth. Plant J. Cell Mol. Biol..

[33] Mehrotra R., Sethi S., Zutshi I., Bhalothia P., Mehrotra S. (2013). Patterns and Evolution of ACGT Repeat Cis-Element Landscape across Four Plant Genomes. BMC Genom..

[34] Liu J., Jiang C., Kang L., Zhang H., Song Y., Zou Z., Zheng W. (2020). Over-Expression of a 14-3-3 Protein From *Foxtail Millet* Improves Plant Tolerance to Salinity Stress in *Arabidopsis thaliana*. Front. Plant Sci..

[35] Schoonheim P.J., Sinnige M.P., Casaretto J.A., Veiga H., Bunney T.D., Quatrano R.S., de Boer A.H. (2007). 14-3-3 Adaptor Proteins Are Intermediates in ABA Signal Transduction during Barley Seed Germination. Plant J. Cell Mol. Biol..

[36] Zhang Y., Zhao H., Zhou S., He Y., Luo Q., Zhang F., Qiu D., Feng J., Wei Q., Chen L. (2018). Expression of *TaGF14b*, a 14-3-3 Adaptor Protein Gene from Wheat, Enhances Drought and Salt Tolerance in Transgenic Tobacco. Planta.

[37] Gupta B., Huang B. (2014). Mechanism of Salinity Tolerance in Plants: Physiological, Biochemical, and Molecular Characterization. Int. J. Genom..

[38] Leng B., Wang X., Yuan F., Zhang H., Lu C., Chen M., Wang B. (2021). Heterologous Expression of the Limonium Bicolor MYB Transcription Factor LbTRY in *Arabidopsis thaliana* Increases Salt Sensitivity by Modifying Root Hair Development and Osmotic Homeostasis. Plant Sci. Int. J. Exp. Plant Biol..

[39] Wu W., Zhang Q., Ervin E.H., Yang Z., Zhang X. (2017). Physiological Mechanism of Enhancing Salt Stress Tolerance of Perennial Ryegrass by 24-Epibrassinolide. Front. Plant Sci..

[40] Qiu Z., Guo J., Zhu A., Zhang L., Zhang M. (2014). Exogenous Jasmonic Acid Can Enhance Tolerance of Wheat Seedlings to Salt Stress. Ecotoxicol. Environ. Saf..

[41] Shi H., Zhang Y. (2014). Pear 14-3-3a Gene (Pp14-3-3a) Is Regulated during Fruit Ripening and Senescense, and Involved in Response to Salicylic Acid and Ethylene Signalling. J. Genet..

[42] Garg N., Bharti A. (2018). Salicylic Acid Improves Arbuscular Mycorrhizal Symbiosis, and Chickpea Growth and Yield by Modulating Carbohydrate Metabolism under Salt Stress. Mycorrhiza.

[43] Ishida S., Fukazawa J., Yuasa T., Takahashi Y. (2004). Involvement of 14-3-3 Signaling Protein Binding in the Functional Regulation of the Transcriptional Activator Repression of Shoot Growth by Gibberellins. Plant Cell.

[44] Maggio A., Barbieri G., Raimondi G., De Pascale S. (2010). Contrasting Effects of GA3 Treatments on Tomato Plants Exposed to Increasing Salinity. J. Plant Growth Regul..

[45] Mao J., Niu C., Li K., Mobeen Tahir M., Khan A., Wang H., Li S., Liang Y., Li G., Yang Z. (2020). Exogenous 6-Benzyladenine Application Affects Root Morphology by Altering Hormone Status and Gene Expression of Developing Lateral Roots in *Malus hupehensis*. Plant Biol..

[46] Hasegawa P.M., Bressan R.A., Zhu J.-K., Bohnert H.J. (2000). Plant Cellular and Molecular Responses to High Salinity. Annu. Rev. Plant Physiol. Plant Mol. Biol..

[47] Blumwald E. (2000). Sodium Transport and Salt Tolerance in Plants. Curr. Opin. Cell Biol..

[48] Shabala S., Pottosin I. (2014). Regulation of Potassium Transport in Plants under Hostile Conditions: Implications for Abiotic and Biotic Stress Tolerance. Physiol. Plant..

[49] Lin S.-M., Tsai J.-Y., Hsiao C.-D., Huang Y.-T., Chiu C.-L., Liu M.-H., Tung J.-Y., Liu T.-H., Pan R.-L., Sun Y.-J. (2012). Crystal Structure of a Membrane-Embedded H^+^-Translocating Pyrophosphatase. Nature.

[50] Zhao C., Zhang H., Song C., Zhu J.-K., Shabala S. (2020). Mechanisms of Plant Responses and Adaptation to Soil Salinity. Innovation.

[51] Poole R.L. (2007). The TAIR Database. Methods Mol. Biol. Clifton NJ.

[52] Chen C., Chen H., Zhang Y., Thomas H.R., Frank M.H., He Y., Xia R. (2020). TBtools: An Integrative Toolkit Developed for Interactive Analyses of Big Biological Data. Mol. Plant.

[53] Bailey T.L., Johnson J., Grant C.E., Noble W.S. (2015). The MEME Suite. Nucleic Acids Res..

[54] Tamura K., Peterson D., Peterson N., Stecher G., Nei M., Kumar S. (2011). MEGA5: Molecular Evolutionary Genetics Analysis Using Maximum Likelihood, Evolutionary Distance, and Maximum Parsimony Methods. Mol. Biol. Evol..

[55] Lescot M., Déhais P., Thijs G., Marchal K., Moreau Y., Van de Peer Y., Rouzé P., Rombauts S. (2002). PlantCARE, a Database of Plant Cis-Acting Regulatory Elements and a Portal to Tools for in Silico Analysis of Promoter Sequences. Nucleic Acids Res..

[56] Hu A., Yang X., Zhu J., Wang X., Liu J., Wang J., Wu H., Zhang H., Zhang H. (2022). Selection and Validation of Appropriate Reference Genes for RT-qPCR Analysis of *Nitraria Sibirica* under Various Abiotic Stresses. BMC Plant Biol..

[57] Livak K.J., Schmittgen T.D. (2001). Analysis of Relative Gene Expression Data Using Real-Time Quantitative PCR and the 2^−ΔΔCT^ Method. Methods.

[58] Iyer K., Bürkle L., Auerbach D., Thaminy S., Dinkel M., Engels K., Stagljar I. (2005). Utilizing the Split-Ubiquitin Membrane Yeast Two-Hybrid System to Identify Protein-Protein Interactions of Integral Membrane Proteins. Sci. STKE Signal Transduct. Knowl. Environ..

